# SiO_2_ Modification of Silicon Carbide Membrane via an Interfacial In Situ Sol–Gel Process for Improved Filtration Performance

**DOI:** 10.3390/membranes13090756

**Published:** 2023-08-24

**Authors:** Shuangjie Shi, Kejie Jian, Minfeng Fang, Jian Guo, Pinhua Rao, Guanghui Li

**Affiliations:** 1 Innovation Centre for Environment and Resources, School of Chemistry and Chemical Engineering, Shanghai University of Engineering Science, 333 Longteng Road, Shanghai 201620, Chinaraopinhua@sues.edu.cn (P.R.); ghli@sues.edu.cn (G.L.); 2 China Petroleum and Chemical Industry Key Laboratory of Silicon Carbide Ceramic Membrane, Shanghai University of Engineering Science, 333 Longteng Road, Shanghai 201620, China; 3 Shandong SiHYFLUX Membrane Technology Co., Ltd., 2252 Yiwangfu North Road, Qingzhou 262500, China; qzsihai@vip.163.com

**Keywords:** silicon carbide membrane, membrane modification, composite membrane, in situ sol–gel, improved performance, microfiltration, ultrafiltration, pore size control

## Abstract

Silicon carbide (SiC) membrane has emerged as a promising class of inorganic ceramic membranes with many advantageous attributes and has been used for a variety of industrial microfiltration (MF) processes. The state-of-the-art industrial manufacturing of SiC membranes based on the particle sintering method can only achieve an average pore size that ranges from 40 nm to a few micrometers, which is still unsatisfactory for ultrafiltration (UF) applications. Thus, the pore size control of SiC membranes remains a focus of continuing study. Herein, we provide an in situ sol–gel modification strategy to tailor the pore size of SiC membranes by a superficial deposition of SiO_2_ onto the membrane surface and membrane pore channels. Our in situ sol–gel modification method is simple and effective. Furthermore, the physical characteristics and the filtration performance of the membrane can easily be controlled by the in situ reaction time. With an optimal in situ reaction time of 30 min, the average pore size of the membrane can be reduced from macropores (400 nm) to mesopores (below 20 nm), and the retention ability for 20 nm fluorescent PS microspheres can be improved from 5% to 93%; the resultant SiC/SiO_2_ composite membranes are imparted with water permeance of 77 L·m^−2^·h^−1^·bar^−1^, improved anti-protein-fouling properties, excellent performance, and anti-acid stabilities. Therefore, modified SiC/SiO_2_ membranes based on the in situ sol–gel process have great potential as UF membranes for a variety of industrial processes.

## 1. Introduction

Silicon carbide (SiC) membrane has emerged as a promising class of inorganic membrane with its many advantageous attributes, such as outstanding chemical and thermal resistance, excellent hydrophilicity, high water permeability, excellent anti-fouling properties, as well as great mechanical properties. Moreover, it has been used for a variety of industrial separation processes such as water treatment, wastewater treatment, food and biotechnology applications, etc. [1,2,3,4]. Currently, commercial SiC membranes are mainly manufactured based on the particle sintering method, where a slurry of SiC particles is deposited on pre-formed macroporous SiC supports and then subjected to high-temperature sintering to form a porous separation layer on top of the support [1,2]. The resulting asymmetric membranes have average pore sizes ranging from 78 nm to a few micrometers, making them suitable for microfiltration (MF) applications [2]. However, the ultrafiltration (UF) application of these commercial SiC membranes is still limited as the average pore size fails to meet the UF requirement (5 to ca. 20 nm). SiC membranes with average pore sizes at the level of nanometers exist, but they are fabricated by using costly, pre-ceramic polymer polycarbosilanes (PCS) at a laboratory scale [5]; other SiC membranes derived from PCS or polytitanocarbosilane (TiPCS) are dense membranes that are suitable for gas separation (GS) [6,7,8,9,10] and pervaporation (PV) [11,12] applications, but are not ideal for water UF applications. Thus, industry-oriented fabrication of SiC membranes with a smaller average pore size that is suitable for UF applications is highly needed.

Different methods have been employed for the fabrication of SiC membranes both in the laboratory and in industry, whereby the pore size and size distribution can be tailored to meet specific filtration requirements [2]. Nevertheless, the particle sintering method dominates the current SiC membrane manufacturing industry, and the control of an average pore size below 20 nm remains challenging; this is because it requires a stringent standard for raw SiC particle materials in terms of particle shape, particle size, and size distribution, which is difficult to provide by the current raw SiC material industry. As a matter of fact, the state-of-the-art SiC membrane that can be manufactured at the industrial level only has an average pore size of 40 nm [13,14].

While the pore size control of SiC membranes remains a focus of continuing study and a challenge to solve [15], an alternative strategy to reduce the pore size and increase the separation selectivity of SiC membranes is to modify the pore channels of existing SiC MF membranes by depositing a suitable porous material on a membrane surface or into pores at a shallow depth underneath the surface. Through this method, the pore channels underneath the surface of the SiC membrane and/or the surface itself will be modified by the porous material, thereby forming a SiC-based composite membrane whose average pore size can be reduced and carefully controlled so as to satisfy UF applications.

In this paper, we aim to modify a commercial SiC MF membrane with SiO_2_ material to form a SiC/SiO_2_ composite membrane and to reduce the average pore size for potential UF applications. The selected commercial SiC membrane is manufactured with a post-oxidation step, where an ultrathin layer of SiO_2_ is formed on the surfaces of exposed SiC particles, as well as on the sintering necks. Surface oxidation is common and advantageous for SiC membranes since the resultant thin layer of SiO_2_ can help prevent further oxidation and can increase hydrophilicity [16,17,18,19,20]. Therefore, the use of SiO_2_ as a modifying material is compatible with the base membrane, and the modification should result in a stable and more hydrophilic composite membrane. In addition, SiO_2_ can be fabricated into a porous material, as evidenced by the fabrication of porous SiO_2_ membranes—both non-supported and supported [21,22,23]. Thus, by modifying SiC membranes with SiO_2_, the resultant SiC/SiO_2_ composite membrane can be imparted with a reduced average pore size and an improved sieving property during filtration while maintaining the advantageous attributes associated with SiC membranes.

The sol–gel process is a very attractive method for the preparation of metal oxide nanoparticles and porous materials due to advantages such as easy control of stoichiometry, thorough mixing at a molecular level, production of ultrafine particles or porous materials, low processing temperature, short processing time, etc. [24,25,26,27,28]. A variety of metal oxide ceramic membranes can be processed by the sol–gel method, including alumina, zirconia, titania, etc. [29]. The sol–gel method has also been utilized extensively for the fabrication of non-metal oxide ceramics, such as carbide ceramics, including SiC [30,31,32,33,34,35,36,37,38,39,40]. Compared to other processing methods, in addition to the aforementioned advantages, the sol–gel process can result in smaller pore sizes and better thieving abilities, and it is a common method for the preparation of ceramic nanofiltration (NF) membranes [14]. In particular, the sol–gel process has been widely applied to fabricate SiO_2_ porous material and ceramic membranes using the precursor tetraethoxysilane (TEOS), and a series of supported SiO_2_ membranes have been reported [41,42,43,44,45]. Nevertheless, the application of the sol–gel process to modify existing SiC membranes with SiO_2_ and to fabricate SiC/SiO_2_ composite membranes seems rare.

Herein, we report a simple and convenient in situ sol–gel process to fabricate a SiC/SiO_2_ composite membrane through the modification of a commercial SiC membrane with SiO_2_. The in situ technique we employ is simple, convenient, effective, and can be easily scaled up at the industry level. The resulting SiC/SiO_2_ membrane demonstrated improved separation performance compared to the pristine SiC membrane, showing reduced average pore size down to the UF region, excellent retention ability, a good combination of permeation and retention properties, excellent anti-protein-fouling properties, as well as performance and chemical stability. This composite membrane has potential application as a UF membrane for a variety of industrial processes.

## 2. Materials and Methods

### 2.1. Materials

Pristine flat-sheet SiC membranes were supplied by Shandong SiHYFLUX Membrane Technology Co., Ltd. (Qingzhou, China). The average pore size, porosity, and water permeance are about 400 nm, 42%, and 8600 L·m^−2^·h^−1^·bar^−1^, respectively. Tetraethoxysilane (TEOS) and nitric acid (65%) of analytical grade were purchased from Shanghai Titan Scientific Co., Ltd. (Shanghai, China). Monodispersed polystyrene (PS) microspheres labeled with fluorescent dyes were purchased from Shanghai Aladdin Biochemical Technology Co., Ltd. (Shanghai, China). Bovine serum albumin (BSA) was purchased from Guangzhou Saiguo Biotech Co., Ltd. (Guangzhou, China). The aqueous solution of phosphate-buffered saline (PBS, pH = 7.4) containing KH_2_PO_4_, KH_2_PO_4_, Na_2_HPO_4_, and NaCl was used as a buffer solution. Deionized water was used throughout the experiment. All other chemicals and reagents were used as received.

### 2.2. Fabrication of SiC/SiO_2_ Composite Membrane via the In Situ SiO_2_ Sol–Gel Process

Prior to the in situ SiO_2_ sol–gel modification, the supplied pristine flat sheet SiC membranes were cut into small membrane coupons (5 cm × 5 cm) and then subjected to ultrasonic cleaning, washing with deionized water and ethanol successively to remove possible impurities remaining on the membrane surface and inside pore channels and, finally, dried at 60 °C for 3 h.

**Figure 1:**
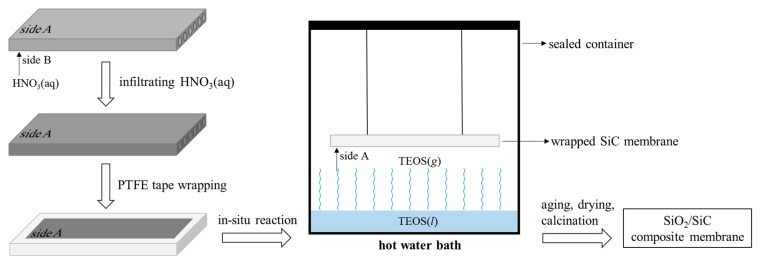
The schematic diagram for the fabrication of SiC/SiO_2_ composite membrane via the in situ sol–gel process.

The in situ SiO_2_ sol–gel modification process of the pristine SiC membrane to fabricate the SiC/SiO_2_ composite membrane is as follows (Figure 1): a membrane coupon is briefly brought in contact with nitric acid with one of its flat surfaces (surface B) so that nitric acid can quickly infiltrate the whole membrane body to reach the other flat surface (surface A) due to capillary force and excellent hydrophilicity of the SiC membrane. The acid-infiltrated SiC membrane was then carefully wrapped with PTFE tape, with only surface A exposed. Subsequently, the wrapped SiC membrane was hung in a closed container over the surface of TEOS liquid, with surface A facing down. The well-sealed container was then immersed in a hot water bath (60 °C) to vaporize TEOS and induce TEOS hydrolysis and condensation on surface A and within the pores near the surface. The extent of the in situ reaction and the depth of the reaction medium into membrane pores can be controlled by reaction time. Once the desired TEOS exposure time was reached, the container was taken out from the bath and cooled to room temperature, and set aside for 24 h to allow the condensation reaction to complete and the SiO_2_ sol to stabilize. After that, the wet gel-modified SiC membrane was dried at 60 °C to remove the remaining solvent and subsequently calcined at 450 °C to form the SiO_2_-modified SiC membrane (SiC/SiO_2_ composite membrane).

### 2.3. Characterization of SiO_2_-Modified SiC (SiC/SiO_2_ Composite) Membrane

Fourier transform infrared (FTIR) spectra were recorded with a Thermo Scientific Nicolet FTIR spectrometer (Nicolet iS20, Waltham, MA, USA) with an ATR accessory in the region of 500–4000 cm^−1^. Scanning electron microscopy (SEM) (Hitachi SU8010, Tokyo, Japan) was used to observe the membrane surface and the cross-section morphological structure after gold sputter-coating samples. Powder X-ray diffraction (XRD) patterns were obtained on a Philips X’PERT MPD diffractometer (Malvern Panalytical Ltd., Almelo, The Netherlands) using monochromatic Cu K*α* radiation (*λ* = 1.5406 Å) at 45 kV and 40 mA. Pore size distribution was measured with a mercury porosimeter (Quantachrome PoreMaster 60, Anton Paar QuantaTec Inc., Boynton Beach, FL, USA) as well as physical adsorption–desorption of N_2_ using a full-automatic specific surface area analyzer (Quantachrome Autosorb IQ3, Anton Paar QuantaTec Inc., Boynton Beach, FL, USA) at 77 K. The samples were prepared by carefully slicing off a thin layer from the membrane top with additional polishing to remove as much remaining SiC support as possible. Prior to N_2_ physical adsorption–desorption measurement, samples were degassed under vacuum at 473 K for 12 h. The pore size distribution was calculated based on density functional theory (DFT) [46]. The surface hydrophilicity of the pristine SiC membrane and the SiC/SiO_2_ composite membranes was estimated on a drop shape analyzer (SDC-200S, Dongguan Sindin Instrument Co., Ltd., Dongguan, China) by measuring the water contact angle based on the drop liquid method at 25.0 °C. The measurement of the water contact angle for each membrane was repeated at least three times.

### 2.4. Fluorescent Polystyrene (PS) Microsphere Retention Test

Fluorescent and water-dispersible polymer nanospheres with uniform diameters can be used as probes to determine the effective pore size of membranes [47]. Monodispersed fluorescent PS microspheres are commercially available and the method to measure pore size using these microspheres has been established [48]. Standard PS fluorescent microspheres with a certain nominal diameter were diluted to 1 μg/mL with deionized water and thoroughly sonicated, and then filtered through the membrane. Subsequently, the fluorescence spectrum of the filtrate was obtained with a fluorescence spectrophotometer (Hitachi F-4700, Tokyo, Japan), and the peak intensity at the corresponding emission wavelength was used to quantify PS microspheres in solution. At a low concentration, the concentration of PS microspheres is directly proportional to the peak intensity at the emission wavelength. The observed solute retention rate (*R*) can be calculated using the following equation:

(1) $$R\left(\%\right)=\left(1-\frac{C_{p}}{C_{f}}\right)\times 100\%$$

where *C*_f_ and *C*_p_ are the concentrations of PS microspheres in feed and permeate, respectively.

All retention tests were conducted at room temperature and the feed solution has a pH of 7.0 ± 0.2. The results presented were average data from at least three samples of each membrane.

### 2.5. Water Permeation Test

**Figure 2:**
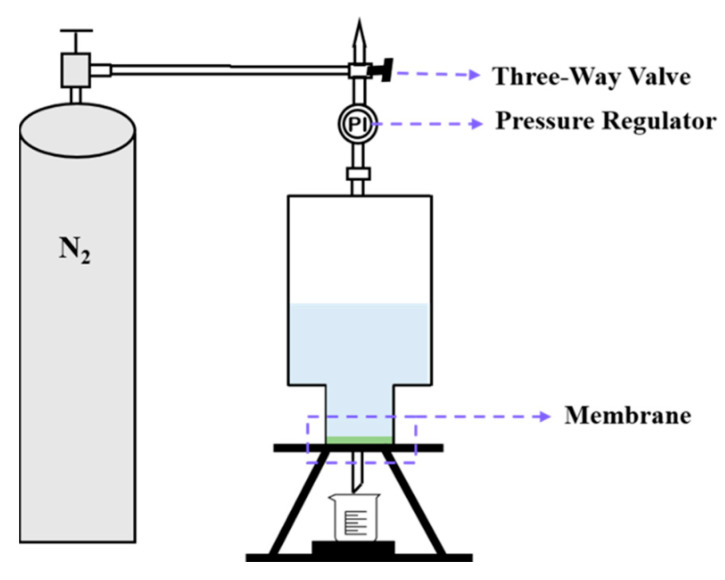
Schematic diagram of the lab-made dead-end water flux measurement setup.

The permeance performance of the membranes was evaluated by using a lab-made dead-end filtration system (Figure 2) and using deionized water as the feed. The permeation flux (*J*_V_) was calculated according to the following equation:

(2) $$J_{V}=\frac{V}{A\times t}$$

where *V* is the total volume of the permeate through the membrane (L), *A* is the effective area of the membrane (m^2^) and *t* denotes the operation time (h).

The pressure-normalized water permeation flux was obtained by linear fitting of the flux value (*J*_V_) to the transmembrane pressure. Independent tests were repeated at least three times with each membrane.

### 2.6. Protein Adsorption and Fouling Experiments

The anti-protein-fouling property of the membrane was evaluated through dynamic fouling experiments using BSA as the model protein via dead-end filtration performed with the above permeation setup [49,50]. To begin with, the filtration was carried out using deionized water as the feed and an initial flux of *J*_w0_ was obtained, which was almost constant for each membrane during the whole experiment except for the washing step. The feed was then replaced by a 50 μg/L BSA solution in PBS solution (pH = 7.4) and the filtration was performed while the permeate flux was monitored and the initial flux for the first 10 mL filtrate was denoted as *J*_ws_. After the flux decreased to a certain extent, the filtration was stopped and the membrane cell was flipped so that the flow direction through the membrane was reversed and the membrane was backwashed with deionized water at a pressure of 2 bar for 20 min, followed by ultrasonic cleaning for 2 min. Finally, deionized water was filtered again through the membrane and the stable water flux was detonated as *J*_wc_. These steps constitute one cycle of the fouling–cleaning operation. Two complete cycles of such operation were performed.

The detected fluxes *J*_w0_, *J*_ws_, and *J*_wc_ were used to evaluate the relative flux decline ratio (RFDR) and the flux recovery ratio (FRR) of the membrane using Formulas (3) and (4), respectively:

(3) $$RFDR=\frac{J_{w0}-J_{ws}}{J_{w0}}\times 100\%$$

(4) $$FRR=\frac{J_{wc}}{J_{w0}}\times 100\%.$$

Higher FRR values and lower RFDR values suggest better anti-protein-fouling ability.

### 2.7. Membrane Stability Test

In order to investigate the physical and chemical stability of the prepared composite membranes, the membranes were subjected to acid treatment, thermal treatment, and backflushing, respectively, and their properties and performance were re-evaluated. A membrane was soaked in a 0.1 M H_2_SO_4_ aqueous solution for 3 h as acid treatment; a membrane was heated in water at 80 °C for 3 h as hydrothermal treatment; for backflushing treatment, a membrane was backflushed with deionized water at 4 bar for 1 h. For each treatment, the membrane was cleaned when necessary and oven dried, and the surface morphology, water permeation performance, and retention of PS microspheres were measured again and compared to that of untreated membranes.

## 3. Results and Discussion

### 3.1. The In Situ SiO_2_ Sol–Gel Modification of the SiC Membrane

**Figure 3:**
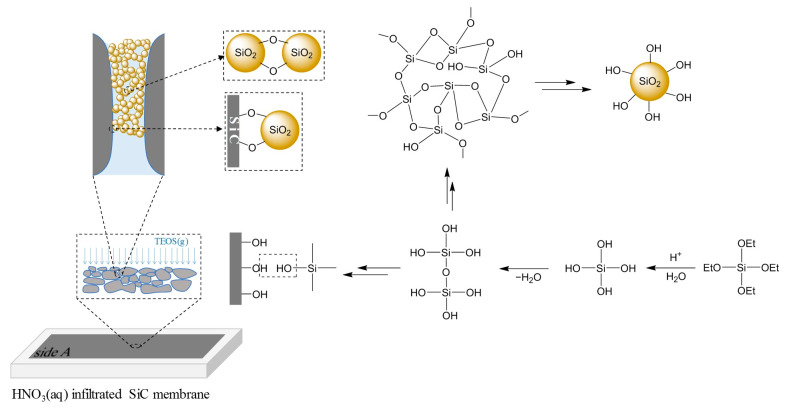
The hydrolysis and condensation of TEOS to form a SiO_2_ sol and the interconnection of SiO_2_ particles as well as the interaction of SiO_2_ particles with the SiC pore wall during the in situ sol–gel process within the pore channels in close vicinity to the membrane surface.

The in situ sol–gel process we employed to modify the pores of the SiC membrane is simple and effective. As shown in Figure 1, the SiC membrane to be modified, after being infiltrated by aqueous HNO_3_, is simply hung above liquid TEOS within a closed container, which is placed in a hot water bath (approx. 60 °C). TEOS starts to evaporate at 45 °C, and the vapor pressure at 60 °C is about 0.02 bar, indicating that the amount of TEOS in the gas phase is small but once it rises and reaches the surface of the SiC membrane, its reaction with aqueous HNO_3_ can take place in a controllable way since TEOS is the limiting reagent and the reaction of TEOS with HNO_3_(*aq*) is very fast. The induced in situ sol–gel process takes place on the membrane surface and inside the membrane pores in close vicinity to the membrane surface, and the hydrolysis of TEOS and subsequent condensation steps to form the SiO_2_ network can be envisaged as in Figure 3. As the condensation reaction proceeds, the continuously growing silica network reaches a point where individual silica particles form, stabilize, and pack in the membrane pores. The surface of the SiC membrane can also be covered with a thin layer of SiO_2_ particles, depending on the amount of HNO_3_(*aq*) present on the surface. The resultant SiO_2_ sol after aging is then placed aside to become a gel, and the subsequent drying and calcination step results in a porous SiO_2_ network inside the pores of the SiC membrane. The depth of SiO_2_ porous material penetrating the SiC membrane can be controlled by the interfacial in situ reaction time, i.e., the exposure time of side A of the membrane to gaseous TEOS. The termination of further reactions of TEOS with HNO_3_(*aq*) can be simply archived by removing the container from the hot water bath and cooling it down. Therefore, the SiC membrane can be modified in this simple and effective way to form a SiC/SiO_2_ composite membrane, and the degree of modification can be controlled by varying the reaction time. In this study, the SiC/SiO_2_ composite membranes with different degrees of SiO_2_ modification are designated as SiC/SiO_2_-X, where X represents reaction time in minutes.

The final resulting porous silica material confined in existing SiC membrane pores consists of SiO_2_ particles interconnecting with each other. The interconnection of SiO_2_ particles could be due to the sintering effect, as well as the oxo-bridging resulting from the condensation of the hydroxy groups, present on the surfaces of the particles during the drying and calcination steps, as depicted in Figure 3. Additionally, the SiO_2_ particles in close vicinity to the wall of the pores could also be chemically bonded to the wall. The reason is that, since the SiC membrane was manufactured with a post-oxidation process, the membrane surface and the pore wall are covered with a very thin layer of SiO_2_ formed. The abundant –OH groups on the membrane surface and the pore wall can thus function as anchor sites for SiO_2_ particles to bind once SiO_2_ particles have formed in the pores, or participate in the polycondensation reaction to be part of the silica network. Therefore, the silica material confined in the pores should be stable and integrated.

**Figure 4:**
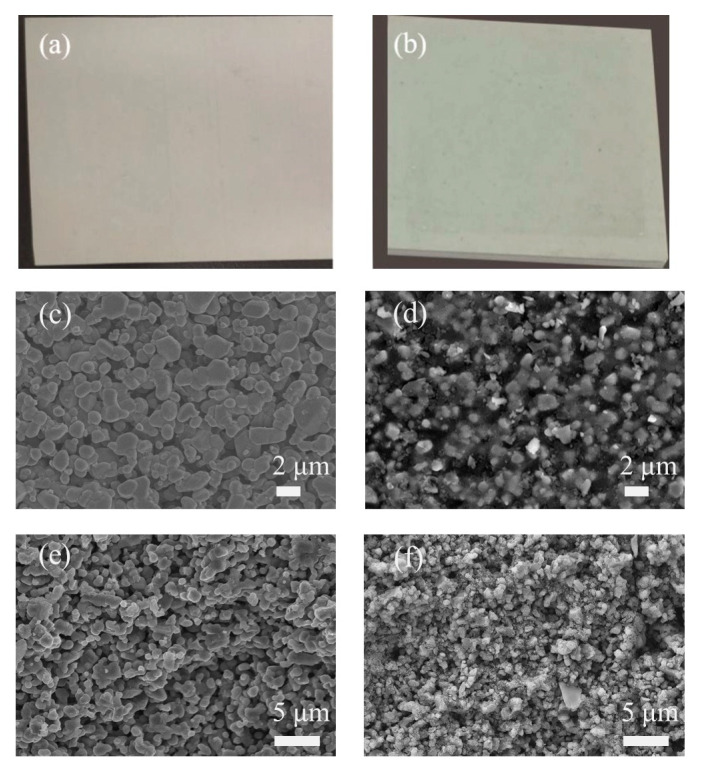
The surface appearance of (**a**) the pristine SiC membrane and (**b**) the SiC/SiO_2_-60 membrane with the naked eye and the corresponding microscopic view with SEM of (**c**) the surface of the pristine SiC membrane, (**d**) the surface of the SiC/SiO_2_-60 membrane, (**e**) the cross-section of the pristine SiC membrane separation layer, and (**f**) the cross-section of the SiC/SiO_2_-60 membrane separation layer.

**Figure 5:**
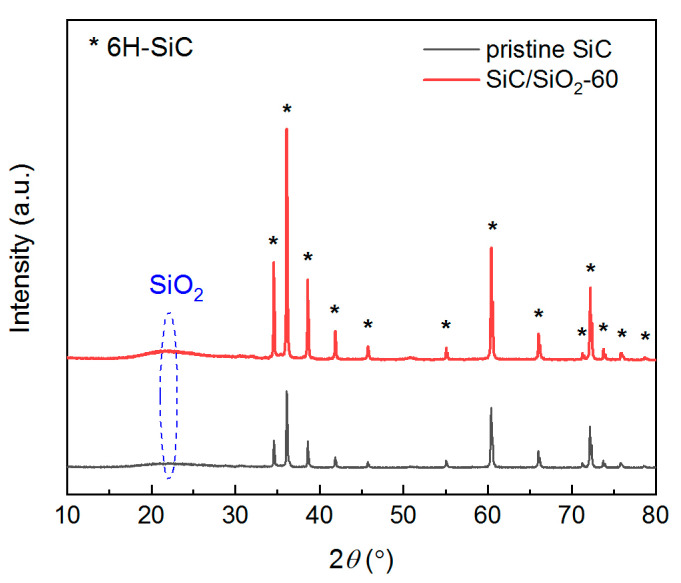
XRD patterns of the pristine SiC membrane and the SiC/SiO_2_-60 composite membrane.

Figure 4 is a comparison of the macroscopic and microscopic views of the SiC membrane before and after the in situ SiO_2_ modification with an in situ reaction time of 60 min. No obvious changes in surface texture can be observed with the naked eye, except for a slight color change. However, in a microscopic view with SEM, they appear to have different morphologies. Surface roughness is greatly reduced after SiO_2_ modification: the interconnected voids between sintered SiC particles are significantly filled by SiO_2_ deposition, as can be seen in Figure 4d. The shape of the sintered SiC particles on the membrane surface also changed from round to sharp, suggesting that a certain degree of SiO_2_ deposition occurred on the membrane surface but not to the extent of forming a separate layer. From the cross-section view of the membrane separation layer, it can be seen that the interconnected pore channels are largely filled by SiO_2_ particles after modification, effectively changing the pore structure of the pristine membrane. To confirm the existence of these SiO_2_ particles, XRD measurements were performed on both the pristine SiC membrane and the modified SiC/SiO_2_-60 composite membrane, and their diffraction patterns were compared, as shown in Figure 5. It can be seen that the XRD patterns for both membranes are very similar: all sharp peaks are due to the strong diffraction of SiC, which is the main component of both membranes, and all can be assigned to 6H-SiC crystalline phase; SiO_2_, on the other hand, exists in an amorphous state or with very small crystal sizes, as only a broad diffuse peak maximized at around 2*θ* = 22° can be observed. This is common for amorphous silica materials or silica nanoparticles [51,52,53]. The existence of SiO_2_ in the pristine SiC membrane is due to the post-oxidation process during manufacturing, as mentioned before. The ratio of the area of the diffuse peak to the total area of 6H-SiC peaks is slightly larger for the SiC/SiO_2_-60 composite membrane than for the pristine SiC membrane, confirming the existence of extra SiO_2_ or SiO_2_ particles on the surface and in the pore channels after membrane modification.

### 3.2. Effect of the In Situ Reaction Time on Membrane Surface Composition and Morphology

**Figure 6:**
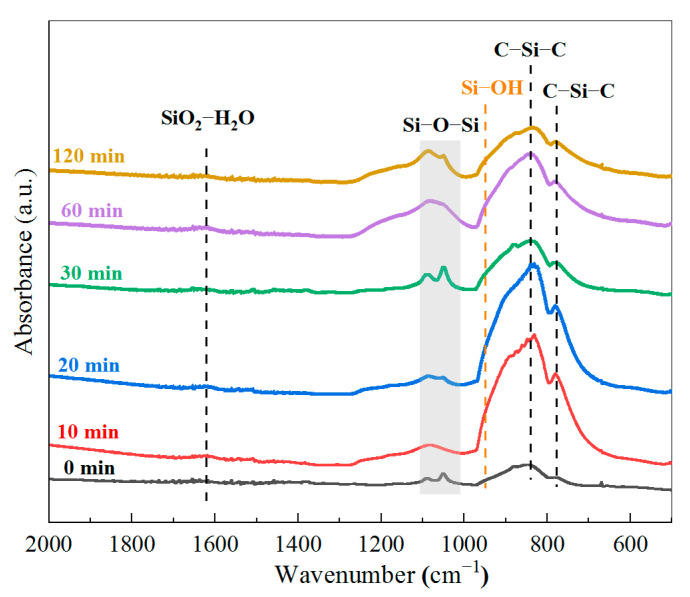
FTIR spectra of SiC/SiO_2_ composite membranes fabricated with different in situ reaction times.

To investigate the relationship of the in situ reaction time with the extent of SiO_2_ modification of the SiC membrane, five pristine SiC membrane coupons were subjected to in situ sol–gel reactions with different reaction times, namely 10, 20, 30, 60, and 120 min, respectively, and IR absorption spectra were collected using an ATR accessory for the five resulting SiC/SiO_2_ composite membranes as well as the pristine SiC membrane, as shown in Figure 6. The intense band at 840 cm^−1^ and a small peak at 780 cm^−1^ are attributed to SiC, which exists in all six samples [54,55]. The band at 1000–1100 cm^−1^ is assigned to the Si–O–Si bridging oxygen stretching [56,57,58], confirming the presence of a SiO_2_ network on the membrane surface. Si–O–Si should also have a stretching band at 810 cm^−1^ [54,57], which, however, is very close to and highly overlapped with the SiC stretching band, thus not easily and directly observed. At 950–960 cm^−1^ is a shoulder of the main peak of 840 cm^−1^ and it is due to the stretching mode of silanol bonds (Si–OH) [54,57,58], which plays an important role in anchoring SiO_2_ particles to the wall of pores through the formation of Si–O–Si bonds (Figure 3). The small peak at around 1620 cm^−1^ indicates the presence of coordinated molecular water within the SiO_2_-structure [56]. A summary of the FTIR data and peak analysis is included in Table 1.

The spectra show that SiO_2_ is also present with the pristine SiC membrane, which is the result of the post-oxidation step during the manufacturing process, as mentioned before, leading to a very thin layer of SiO_2_ on the SiC surface. It can be observed that with the increase in the in situ reaction time during the SiO_2_ modification, the absorbance intensity of the Si–O–Si stretching band gradually increases, indicating that an increased amount of SiO_2_ is formed on the membrane when the membrane is subjected to a longer reaction time. This, in turn, will alter membrane surface morphology.

**Figure 7:**
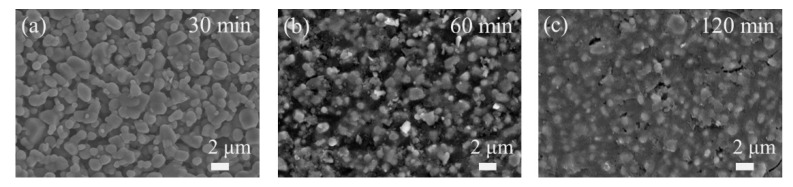
Surface SEM micrographs of SiC/SiO_2_ composite membranes fabricated with different in situ reaction times: (**a**) SiC/SiO_2_-30, (**b**) SiC/SiO_2_-60, and (**c**) SiC/SiO_2_-120.

To investigate the surface morphology of these resultant composite membranes, SEM micrographs of composite membranes with different in situ reaction times were obtained and compared. Figure 7 is a comparison of surface SEM images of three composite membranes with an in situ reaction time of 30, 60, and 120 min, respectively. The surface morphology of SiC/SiO_2_-30 resembles that of the pristine SiC membrane (Figure 4), with a limited amount of SiO_2_ deposited on the surface, and the majority of formed SiO_2_ particles should be confined in the interconnected pore channels near the surface; when the in situ reaction time is increased to 60 min, it reaches an extent that the voids between the SiC sintered particles on the surface (as seen in Figure 7a) are filled with SiO_2_ deposition, with only a small amount of SiO_2_ covering surface SiC particles. The shape of surface SiC particles is, however, still clearly distinguishable from each other, indicating that coverage of SiO_2_ on the surface is avoided (Figure 7b) and that 60 min is appropriate to modify pore channels right to the opening from a certain depth underneath the surface, in accordance to the modified pore structure envisaged in Figure 3, successfully shrinking pore size. However, when the in situ reaction time is 120 min, an excessive amount of SiO_2_ is deposited and the surface is covered with a thin layer of SiO_2_ deposition (Figure 7c). Although some pinholes are present, the majority of the surface is a SiO_2_ network and it would significantly reduce water flux and thus should be avoided.

### 3.3. Effect of the In Situ Reaction Time on the Pore Size and the Pore Size Distribution

**Figure 8:**
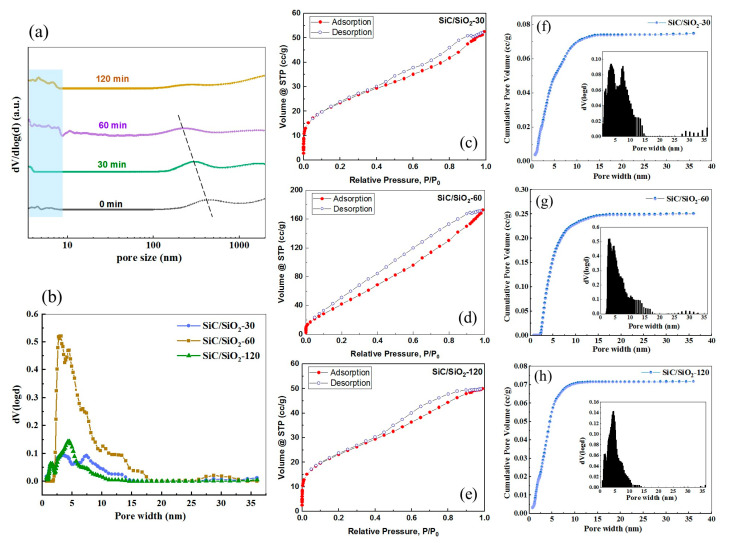
Pore size distribution of SiC/SiO_2_ composite membranes with different in situ reaction times of 30, 60, and 120 min: (**a**) obtained by the mercury porosimetry method and (**b**) obtained by the N_2_ adsorption–desorption method, with the corresponding (**c**–**e**) isotherms of N_2_ adsorption–desorption at 77 K and (**f**–**h**) cumulative pore volume and pore size histogram based on DFT calculation.

In order to investigate the pore size and the pore size distribution changes of the composite membranes brought about by the in situ modification strategy, mercury porosimetry was first employed. Figure 8a shows the pore size distribution for three composite membranes with an in situ reaction time of 30, 60, and 120 min, as well as the pristine SiC membrane. The average pore size of the pristine SiC membrane is about 400 nm according to the supplier’s specification, and this is verified by the wide peak centered at around 400 nm. Pores of diameter less than 10 nm are also present in pristine SiC membranes but only in small fractions, as the peak area is very small compared to that of the large wide peak at 400 nm. As the in situ reaction increases, there is an apparent shift of the broad peak to the left, suggesting that the pore channels of the pristine SiC membrane were successfully modified and reduced, and the extent of reduction increases with the increasing reaction time. Within the <10 nm range, with the increase in reaction time, more micropores are emerging, as is evident from the broadening and rise of the peaks. These indicate that with the increase in the in situ reaction time, more SiO_2_ particles are formed and begin to pack inside the SiC pore channels, eventually reducing the pore volume and pore size with the advent of micropores. When the reaction time reaches 120 min, the curve of 100–400 nm becomes almost flat, indicating that the pore volume of the modified SiC/SiO_2_-120 membrane becomes very small due to extensive modification, which is not benign for membrane application, as the porosity is very low. This is consistent with the SEM results that the SiC/SiO_2_-120 membrane has an excessive amount of SiO_2_ deposited.

N_2_ adsorption–desorption porosimetry was also employed to investigate the pore size distribution of SiC/SiO_2_ composite membranes in microporous and mesoporous regions (<50 nm) and the results are depicted in Figure 8b–h. As can be seen from Figure 8c–e, the isotherms of all SiC/SiO_2_ composite membranes have a capillary condensation hysteresis loop that can be classified as type IV according to the IUPAC classification [59] and are characteristic of pores in the mesoporous region. The pore size distribution was calculated from the adsorption isotherms by applying the DFT method, as it allows the determination of pore size distribution in both the mesoporous and microporous regions [46]. As shown in Figure 8b, both SiC/SiO_2_-30 and SiC/SiO_2_-60 only have mesopores of about three types: most are between 2–10 nm in size, some have sizes between 10–18 nm, and a very small portion around 30 nm. The SiC/SiO_2_-120 membrane, on the other hand, has the presence of micropores in addition to mesopores, as evidenced by the small peak below 2 nm. The mesopores of the SiC/SiO_2_-120 membrane are in the range of 2 to 12 nm and very few peaks around 30 nm in size, suggesting a higher degree of modification compared to that of the other two membranes. All these data confirm the effectiveness of the in situ modification strategy in modifying the pore size and the pore size distribution, and there is a direct correlation between the reaction time and the extent of modification. These results are consistent with those of the mercury porosimetry investigation.

### 3.4. Effect of the In Situ Reaction Time on Membrane Filtration Performance

**Figure 9:**
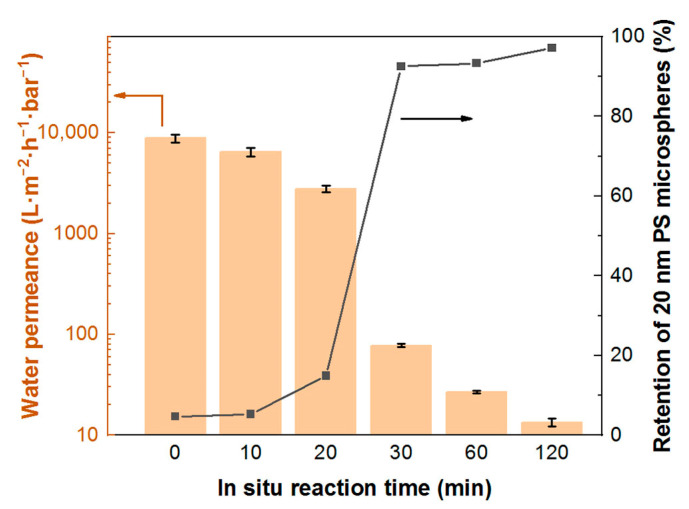
Water permeance and PS microsphere retention properties of SiC/SiO_2_ composite membranes modified with different in situ reaction times.

The results from pore size distribution measurements suggest that the in situ modification of the SiC membrane is effective in reducing the pore size, and the reduction extent is proportional to the reaction time. To evaluate the filtration performance of the composite membranes in terms of water permeation and foulant rejection ability, a water filtration test and a PS microsphere retention test were performed. Figure 9 shows the combined results of water permeation and retention properties of membranes as a function of the in situ reaction time. The pristine SiC membrane has an extremely high water permeance of 8800 L·m^−2^·h^−1^·bar^−1^ due to the large average pore size (400 nm), high porosity (42%) and excellent hydrophilicity. Since the aim of this study was to modify the existing SiC MF membranes for UF applications, fluorescent PS microspheres with a uniform diameter of 20 nm were used as probes to analyze the effective average pore size of the modified composite membranes. The retention rate of 20 nm PS microspheres by the pristine SiC membrane is only 5%, suggesting that the pristine SiC membrane is only good for MF but not for UF applications. When the in situ SiO_2_ sol–gel modification is applied to the pristine membrane, the retention rate of 20 nm PS microspheres begins to increase, reaching 15% with an in situ reaction time of 20 min, and 93% at 30 min; meanwhile, as a “trade-off” effect, water permeance starts to decrease, being 2800 L·m^−2^·h^−1^·bar^−1^ at 20 min and 77 L·m^−2^·h^−1^·bar^−1^ at 30 min. From 20 min to 30 min, there is a sharp jump in retention rate along with a rapid decrease in water permeance, suggesting that a transition change in the pore structure took place during the in situ modification between 20 and 30 min. At the in situ reaction time of 30 min, the average pore size could be considered less than 20 nm, since it can successfully reject more than 90% of PS microspheres with a diameter of 20 nm. As the in situ reaction time increases further, there is only a slight improvement in retention ability, as shown by the flattening of the retention curve after 30 min, but the water permeance drastically decreases further, reaching only 13 L·m^−2^·h^−1^·bar^−1^ at 120 min. From an application point of view, 30 min is an appropriate in situ reaction time with our membrane modification strategy to improve the filtration performance of the pristine membrane for potential UF filtration.

Based on the filtration performance of the SiC/SiO_2_-30 composite membrane and its surface morphology (Figure 7), it can be concluded that the effective in situ modification is due to the packing of the SiO_2_ particles formed inside the interconnected pores rather than on the surface, as there is no apparent SiO_2_ coverage on the surface from the SEM image. The interconnection of the SiO_2_ particles and the connection of SiO_2_ to the wall of pore channels successfully reduce the pore size. However, from the mercury intrusion porosimetry results (Figure 9), the SiC/SiO_2_-30 composite membrane has a pore distribution of around 300 nm, as compared to the average pore size of less than 20 nm from the PS microsphere retention test. This can be interpreted by the fact that the pore size in mercury porosimetry is calculated on the basis of the measured pore volume and a simplification of the pore shape to cylindrical ones and, thus, may deviate from the true values, since it is the narrowest diameter in pore channels that determines the retention ability of the membrane, whilst the majority of the pore structure could have a much larger diameter. We believe that not all interconnected pore channels underneath the membrane surface have been modified by SiO_2_ particles, but only those in regions in close proximity to the surface have undergone modification, and pore channels in greater depth remain intact, so that when under mercury porosimetry measurement the measured pore volume is still large and, hence, the calculated pore size is bigger than that measured by the PS microspheres retention test, which is only determined by the pore structure in the top modified region. This also shows that the fluorescent PS microsphere retention test is a more accurate and practical method for determining the retention ability of membranes than mercury intrusion porosimetry.

### 3.5. Anti-Protein-Fouling Property of SiC/SiO_2_ Composite Membranes

**Figure 10:**
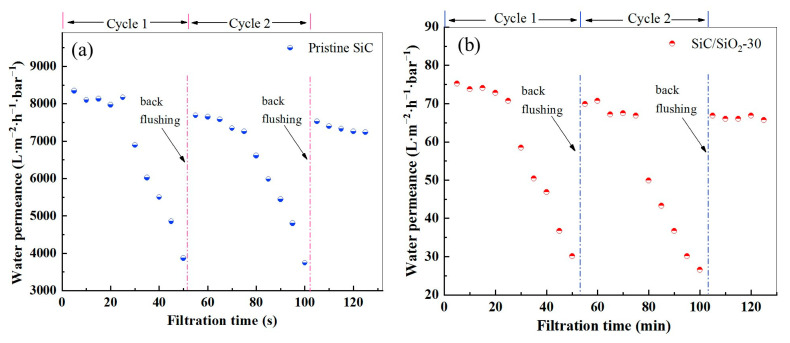
Water permeance of (**a**) the pristine SiC membrane and (**b**) the SiC/SiO_2_-30 composite membrane during a dynamic two-cycle fouling test performed with 50 μg/L BSA aqueous solution.

The anti-fouling ability is crucial for membranes in separation operation as it affects the flux of permeate and the lifetime of membranes. During practical application, foulants, such as proteins, can adsorb onto the membrane surface through hydrophobic interaction, hydrogen bonding, van Der Waals attraction, and electrostatic interaction [60]. Therefore, an effective method to reduce membrane fouling is to improve membrane anti-adsorption ability by minimizing these adsorptive interactions by increasing the hydrophilicity of the membrane surface. SiC itself is a hydrophilic material, and as mentioned before, the pristine SiC membrane is fabricated with a post-oxidation step, so the surface of sintered particles is covered with a thin layer of SiO_2_, which renders higher hydrophilicity compared to that of SiC itself due to the hydroxyl groups present on the surface of the SiO_2_ network (Figure 6). The modified SiC/SiO_2_ composite membrane is expected to be more hydrophilic compared to the pristine SiC membrane due to the abundance of hydroxyl groups associated with the deposited SiO_2_ in the pore channels and on the membrane surface; thus, the SiC/SiO_2_ composite membrane should have better anti-protein-fouling ability. To verify this, the anti-protein-fouling ability of the pristine SiC membrane and the SiC/SiO_2_-30 composite membrane was compared by a dynamic dead-end BSA fouling experiment. During the experiment, a 50 μg/L BSA aqueous solution was filtered through the membranes to induce fouling; once the flux decreased to a certain value, the membrane was subjected to backwashing, and a second filtration cycle was started. The time-dependent water permeance is shown in Figure 10.

As can be seen in Figure 10, in each cycle, due to the clogging of BSA molecules in the membrane pores and the formation of a BSA cake layer on the membrane surface, the water permeance for both membranes decreases steadily and then rapidly as the filtration proceeds. After a certain filtration time, backwashing can restore water permeance to a certain extent, and the trend continues for a second cycle. To evaluate and compare the anti-protein-fouling ability of the pristine SiC membrane and the modified membrane, the RFDR and FRR values are calculated from the flux data and are summarized in Table 2.

In general, a lower RFDR means better resistance to protein adsorption and a higher FRR means better physical cleaning efficiency. Hence, both the lower value of RFDR and the higher value of FRR indicate better anti-protein-fouling ability. For both membranes, anti-protein-fouling ability decreases in cycle 2 compared to that in cycle 1, as suggested by the increased RFDR and the decreased FRR values, due to the irreversible protein fouling taking place during the BSA solution filtration process. When comparing the pristine SiC and the SiC/SiO_2_-30 composite membranes, the latter demonstrates better anti-protein-fouling ability, as is evident from the lower RFDR values in both cycles (2.3% vs. 5.8% and 9.3% vs. 13.3% in cycle 1 and cycle 2, respectively) and the higher FRR values (98% vs. 94% and 91% vs. 86% in cycle 1 and cycle 2, respectively). These results confirm the idea that the introduction of SiO_2_ to the pristine SiC membrane through the in situ modification improves the hydrophilicity of the original membrane thanks to the abundant hydroxy groups present on the exposed SiO_2_ surface.

### 3.6. Performance and Chemical Stability of SiC/SiO_2_ Composite Membranes

**Figure 11:**
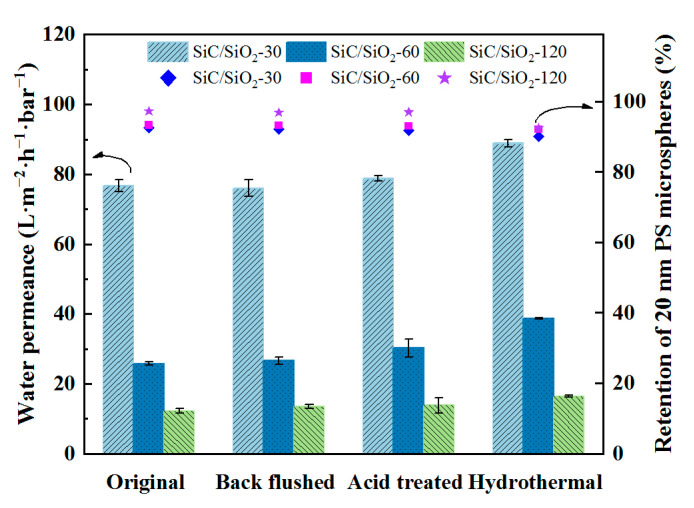
Effect of different treatments on water permeation properties and PS microsphere’s retention ability of three composite membranes fabricated with different in situ reaction times.

Membrane stability is also an important factor in evaluating the performance of a membrane in practical applications. To test the stability of the modified membranes, three treatments were applied to the composite membranes: backflushing, acid treatment, and hydrothermal treatment; water permeation property and PS microsphere retention ability were then evaluated for membranes after treatment. As shown in Figure 11, the water permeance and retention rate of 20 nm PS microspheres of all three composite membranes underwent no apparent changes after backflushing and acid treatment, suggesting that the composite membranes have excellent performance and anti-acid corrosion stabilities. While under hydrothermal conditions, the composite membranes have a small drop in retention ability associated with a small increase in water permeance, indicating a structural change in the pore channels after hydrothermal treatment at 80 °C, possibly due to the loss of SiO_2_ particles within the pore channels under such conditions. Nevertheless, the SiC/SiO_2_ composite membranes demonstrate excellent performance and chemical stabilities under ambient conditions.

## 4. Conclusions

In order to reduce the average pore size of existing SiC MF membranes and improve the retention ability for UF applications, we have employed a simple, convenient, and effective interfacial in situ sol–gel process to modify a commercial SiC flat-sheet membrane with SiO_2_ and to fabricate a SiC/SiO_2_ composite membrane. SEM and FTIR measurements confirm the successful deposition and modification of the pristine SiC membrane by the observed changes in composition and morphology. The pore size distribution data from mercury intrusion porosimetry and N_2_ adsorption–desorption and the average pore size measured by the PS microspheres retention method indicate the effectiveness of pore size modification by the in situ sol–gel process. The surface morphology, water permeation property, and retention ability of PS microspheres can be tailored by varying the in situ reaction time. With an in situ reaction time of 30 min, the SiC/SiO_2_ composite membrane has an average pore size below 20 nm and can reject 93% of 20 nm PS microspheres, with a water permeance of 77 L·m^−2^·h^−1^·bar^−1^. The modified composite membranes also have improved anti-protein-fouling properties compared to pristine membranes, excellent performance, and anti-acid-corrosion stability. Our in situ sol–gel modification strategy is convenient and effective and can be scaled up at the industrial level to modify existing SiC MF membranes for a variety of UF applications.

## Figures and Tables

**Table 1:** FTIR peak assignment for SiC/SiO_2_ composite membranes.

Wavenumber (cm^−1^)	780	810	840	950–960	1000–1100	1620
vibration type	stretching	stretching	stretching	stretching	stretching	stretching
peak intensity	medium	not seen (overlapped)	strong	weak (shoulder)	medium	weak
assignment	Si−C	Si−O−Si	Si−C	Si−OH	Si−O−Si	SiO_2_−H_2_O

**Table 2:** RFDR and FRR values of the pristine SiC and the SiC/SiO_2_-30 membranes during a two-cycle BSA fouling test.

Membranes	Cycle 1		Cycle 2	
	RFDR	FRR	RFDR	FRR
pristine SiC	5.8%	94%	13.3%	86%
SiC/SiO_2_-30	2.3%	98%	9.3%	91%
